# Modeling the potential impacts of automated vehicles on pollutant emissions under different scenarios of a test track

**DOI:** 10.1186/s40068-022-00276-2

**Published:** 2022-12-12

**Authors:** Zelalem Birhanu Biramo, Anteneh Afework Mekonnen

**Affiliations:** 1 Faculty of Civil Engineering, Arba Minch Institute of Technology, Arba Minch University, P.O.Box 21, Arba Minch, Ethiopia; 2 Department of Transport Technology and Economics, Faculty of Transportation Engineering and Vehicle Engineering, Budapest University of Technology and Economics, Muegyetem Rkp. 3, Budapest, 1111 Hungary; 3 School of Civil and Environmental Engineering, Addis Ababa Institute of Technology, Addis Ababa University, P.O. Box 385, Addis Ababa, Ethiopia

**Keywords:** Air pollution, Automated vehicles, Fuel consumption, Pollutant emissions, SUMO

## Abstract

One of the significant sources of air pollution and greenhouse gas emissions is the road transportation sector. These emissions are worsened by driving behaviors and network conditions. It is common knowledge that experienced and inexperienced drivers behave differently when operating vehicles. Given the same vehicle in a different timeframe, the drivers’ reactions to similar situations vary, which has a significant influence on the emissions and fuel consumption as their use of acceleration and speed differ. Because the driving patterns of automated vehicles are programmable and provide a platform for smooth driving situations, it is predicted that deploying them might potentially reduce fuel consumption, particularly in urban areas with given traffic situations. This study’s goal is to examine how different degrees of automated vehicles behave when it comes to emissions and how accelerations affect that behavior. Furthermore, the total aggregated emissions on the synthesized urban network are evaluated and compared to legacy vehicles. The emission measuring model is based on the Handbook Emission Factors for Road Transport (HBEFA)3 and is utilized with the Simulation of Urban Mobility (SUMO) microscopic simulation software. The results demonstrate that acceleration value is strongly correlated with individual vehicle emissions. Although the ability of automated vehicles (AVs) to swiftly achieve higher acceleration values has an adverse effect on emissions reduction, it was compensated by the rate of accelerations, which decreases as the automation level increases. According to the simulation results, automated vehicles can reduce carbon monoxide (CO) emissions by 38.56%, carbon dioxide (CO_2_) emissions by 17.09%, hydrocarbons (HC) emissions by 36.3%, particulate matter (PM_x_) emissions by 28.12%, nitrogen oxides (NO_x_) emissions by 19.78% in the most optimistic scenario (that is, when all vehicles are replaced by the upper bound automated vehicles) in the network level.

## Background

More than 4.2 million lives are expected to have been lost because of ambient air pollution. The World Health Organization (WHO) estimates that 99% of people worldwide reside in areas where air pollution is above WHO’s standard in the revised guidelines. The organization has never revised the air pollution rules since the initial document was issued in 2005. Despite the fact that nearly all nations fell short of the previous standard, WHO is pushing nations to significantly cut emissions in order to meet the ambitious goal of 80% reduction in the number of fatalities brought on by air pollution (Ambient (outdoor) air pollution 2022; World Health Organization 2021). According to a study by the Swiss air quality technology company, 97% of all countries and cities worldwide failed to achieve the most recent WHO PM_2.5_ air quality standard guidelines of 2021 (Empowering the World to Breathe Cleaner Air 2022). Additionally, researchers at the Harvard University T.H. Chan School of Public Health discovered that higher concentrations of the tiny, dangerous airborne particles known as PM_2.5_ were connected to higher death rates from diseases like COVID-19 in an analysis of 3080 counties in the United States of America (USA) (Friedman 2020).

In addition to the common air pollutants that WHO has identified as being particularly dangerous to human health (particulate matter, ozone, nitrogen dioxide, sulfur dioxide, and carbon monoxide), greenhouse gases also have adverse effects on human health in addition to causing climate change and global warming. The human body can tolerate short-term exposure to these gases, but long-term exposure to high concentrations of these gases steadily damages several organs, including the respiratory, cardiovascular, central nervous, immune, digestive, and reproductive systems (Naiyer and Abbas 2022; Jacobson et al. 2019).

Road transport emissions are the principal sources of greenhouse gases and the leading cause of air pollution (Chapman 2007; Albuquerque et al. 2020). These emissions are exacerbated by driving behaviors and network conditions. Rather than how far a vehicle has travelled, the driving style of a vehicle has a significant impact on exhaust emissions. Most literature revealed that the quantity of emissions per vehicle is largely determined by the driving style and conditions of the road, though vehicle design also plays a role. It is well known that experienced and inexperienced drivers operate vehicles in different ways. The drivers’ responses to similar situations when operating the same vehicle at different timeframe varies, and this has a big impact on emissions and fuel consumption because of how differently they use acceleration and speed (Brundell-Freij and Ericsson 2005; Frey et al. 2002). Similar to this, network conditions such as congestion and stop-and-go traffic have the potential to adversely affect aggregate emissions (Barth and Boriboonsomsin 2008; Aminzadegan et al. 2022). However, throughout the past two decades, numerous initiatives have been taken to reduce vehicle emissions (Shaheen and Lipman 2007). These attempts involve modifying vehicle designs to increase combustion engine efficiency (Reitz and Duraisamy 2015), transitioning toward electric and hybrid vehicles (Fontaras et al. 2008), trying to raise awareness of eco-friendly driving (Coloma et al. 2020), encouraging the use of public, soft mobility, and shared transportation alternatives instead of private motor vehicles (Nelldal and Andersson 2012). Solutions for managing traffic networks have been worked on extensively to prevent congestion, which has a considerable impact on emissions (Eco-Cooperative Adaptive Cruise Control at Signalized Intersections Considering Queue Effects 2022). Given the driving scenarios of automated vehicles, it is anticipated that their implementation might substantially reduce fuel consumption, particularly in urban areas (Chen et al. 2019). However, it is still unclear whether automated vehicles would result in an increase or decrease in the overall emissions from road traffic.

A notable assertion with respect to automated vehicles is the Jevons paradox, which states that automated vehicles may increase travel demand, resulting in a significant increase in vehicle kilometers travelled and vehicle hours travelled, as well as a decrease in public transportation mode share (Soteropoulos et al. 2019). Despite the paradox, AVs have the potential to lower fuel consumption and emissions by enabling vehicles to operate more efficiently. It may reduce the amount of fuel wasted when vehicles become trapped in a traffic jam by enhancing traffic flow and lowering accident frequency (ScienceDaily 2022). Vehicle automation may accelerate the widespread adoption of eco-driving, which is a set of practices aimed at reducing fuel consumption without modifying the vehicle architecture. Eco-driving is achieved by either optimizing engine performance or reducing the frequency of a vehicle’s recurring braking and acceleration cycles. According to studies, even human drivers who receive real-time eco-driving instruction can lower their fuel use by 10–20% (Barth and Boriboonsomsin 2009). Similarly, in congested conditions, it may lower fuel consumption by 35–50% (He et al. 2012). In addition, 90% of accidents are typically linked to human error. Automation has the potential to significantly reduce accident rates by minimizing human intervention in driving tasks, so vehicle safety features will become far less relevant in the future. It seems implausible to remove the safety features, but just doing so reduces fuel usage by 5.5% (MacKenzie 1975; Kopelias et al. 2020). As a result, it is anticipated that AVs will cut down emissions from the road traffic sector.

Several models have been developed to evaluate the emissions of individual vehicles to examine the effects of AVs on road traffic emissions and fuel consumption. Previously, direct emission measurement was used, and extensive research was conducted in those areas (Harrington 1997; Frey et al. 2003; Chen et al. 2018). However, because of the inaccessibility of emission-measuring equipment, major research has been done to develop emission-measuring models that simply require a distance and speed profile to predict emission quantities (Pelkmans and Debal 2006; Rakha et al. 2004; An et al. 1997; Giechaskiel et al. 2018). Most of the models used to estimate emissions were developed in the laboratory using chassis dynamometer data from supposedly representative vehicles. These models can estimate typical vehicle emissions pollutants.

In general, emission models are divided into two categories, namely aggregate models, and microscopic models. When planning a new project, the environmental impact is assessed using aggregate models, which calculate emissions based on inputs such as average link-level speed and travel distance. These models are frequently useful for understanding how a significant change in land use and transportation patterns may affect local or regional emissions. The most common aggregate models in Europe are COPERT (Computer Program to Calculate Emissions from Road Transport) and ARTEMIS (Assessment and Reliability of Transport Emission Models and Inventory Systems), while MOVES (Motor Vehicle Emissions Simulator) is in the USA (André et al. 2009; European Environment Agency 2022; O. US EPA 2016). On the other hand, microscopic emissions models calculate emissions for a specific trip using real-time data collected at the vehicle level. In Europe and USA, the most widely used microscopic emission models are PHEM/HBEFA and MOVES (used for both aggregate and microscopic models), respectively.

The HBEFA was established on behalf of environmental protection organizations in Germany, Austria, and Switzerland. HBEFA is supported by other countries (Sweden, Norway, and France), as well as the JRC (Joint Research Centre of the European Commission). The original data were collected and analyzed from several research projects by the Technical University of Graz (Austria), which used the Passenger Car and Heavy-Duty Emission Model (PHEM4) to develop the emission factors. The initial version (HBEFA 1.1) was launched in December 1995, and an update (HBEFA 1.2) was made publicly available in January 1999. The release of HBEFA 2.1 was in February 2004. HBEFA 3.2, the most recent upgrade, was made available in January 2014. The ERMES group, which also provides the emission database for other European models like COPERT, is the source of the data employed by HBEFA to estimate European emissions. HBEFA provides pollution factors, or emissions in grams per kilometer, for all existing vehicle types (passenger cars, light-duty vehicles, heavy-duty vehicles, urban buses, coaches, and motorbikes), each categorized into various size categories and for a variety of traffic situations. Each traffic situation is characterized by a typical driving pattern, which is a sequence of data points showing the speed of a vehicle versus time.

The goal of this research is thus to assess the effectiveness of automated vehicles in reducing air pollution by developing models of AVs with varying degrees of automation and gradually introducing them into the network at increasing market penetration rates. Legacy vehicles, in general, will be used as a baseline model for comparing emission reductions achieved by the deployment of automated vehicles into the network. To carry out simulations and analyze the results, we modeled all legacy and automated vehicles using the Simulation of Urban Mobility software (SUMO). The article’s main objective is to estimate legacy vehicles’ emissions and monitor the emission changes that might happen due to the gradual deployment of automated vehicles on the road. Although, it is evident that weather and road conditions affect the vehicles’ operational behavior (Pappalardo et al. 2022), it’s represented in our simulation as drivers’ imperfection parameter under any circumstances including weather condition and road characteristics. The specific objectives are defining the emission behaviors across various types of vehicles and quantifying the overall influence of vehicle automation on network-level emissions.

Reviews and summaries of related studies have shown that there is an interconnection between driving behaviors and emissions. A study looked at the effects of smooth driving behavior on emissions reductions, which could be achievable by AVs. The driving profile for the AVs is developed by modifying the speed profile of the conventional vehicle by employing smoothing techniques. The MOVES model from the EPA was used to calculate emissions for both conventional and AVs driving profiles. The findings suggested that the usage of AVs could potentially result in lower emissions. In contrast to our current work, they replaced legacy vehicles with AVs rather than considering how differing AVs fleet market penetration rates influence emissions. Furthermore, the levels of vehicle automation were not taken into account (Liu et al. 2017).

Another study looked at the traffic efficiency and environmental implications of AVs at market penetration rates of 10, 20, and 30% along metropolitan motorway corridors. They used PTV Vissim microsimulation with vehicle-specific power (VSP/EPA) and emission methodologies (EMEP/EEA) to estimate emissions. They measured the amounts of CO_2_, CO, NOx, and HC as well as travel time and stop-and-go situations. Their findings revealed that the overall emission had not reduced much; for instance, with 30% AVs, the number of stops had reduced by 15% and the average emission reduction had been only 3%. They chose to focus on only two vehicle types: conventional vehicles (CV) and (AVs), excluding the varying levels of automation, which the current study considers. No measurement was done on VOCS, fuel consumption, or particulate matter (Tomás et al. 2020).

Furthermore, these researchers looked at how connected vehicles and AVs alter the fuel consumption and emissions of mixed traffic flows on the expressway. The researchers employed three car-following models to properly describe car-following behaviors in the mixed traffic flow. A numerical simulation has been developed to investigate how connected vehicles and AVs impact fuel consumption and emissions in mixed traffic flows. The results revealed that connected automated vehicles (CAV) can significantly reduce transportation fuel consumption and emissions. At a 100% penetration rate of CAV, the highest reduction percentages of HC, NOx, CO, and fuel usage are 24.33%, 27.06%, 37.53%, and 40.58%, respectively (Yao et al. 2021).

Most earlier studies had the same shortcomings, failing to account for the different levels of automation in automated vehicles and completely precluding the reality that the automation of the transportation sector is a slow process. The novelty of the current study lies in attempting to bridge the gaps pointed out thus far by modeling varying levels of AVs and gradually deploying them into the network than considering AVs in general as most of the previous studies reviewed above had done. Moreover, according to the researchers’ knowledge and database investigation, this is the first study in the context of AVs impact on road traffic emission in the case of ZalaZone test track.

This paper is organized as follows: The goals and objectives are introduced in the first chapter, along with a review of related studies that have been conducted in the past. “Materials and methods” section describes the car-following model and the corresponding parameters used to model varying levels of AVs, network setup, market penetration rate, emission methods, and simulation trials (including demand profile). “Results and discussions” section discusses the primary outcomes of the simulation experiments while concluding remarks are provided in “Conclusion” section.

## Materials and methods

### Description of the study area

Since the impact of road traffic emissions is less noticeable in rural areas, this study focuses on urban networks. To better portray the current and future urban environment, ZalaZone has been chosen to study and assess the impact of road traffic.

**Fig. 1:**
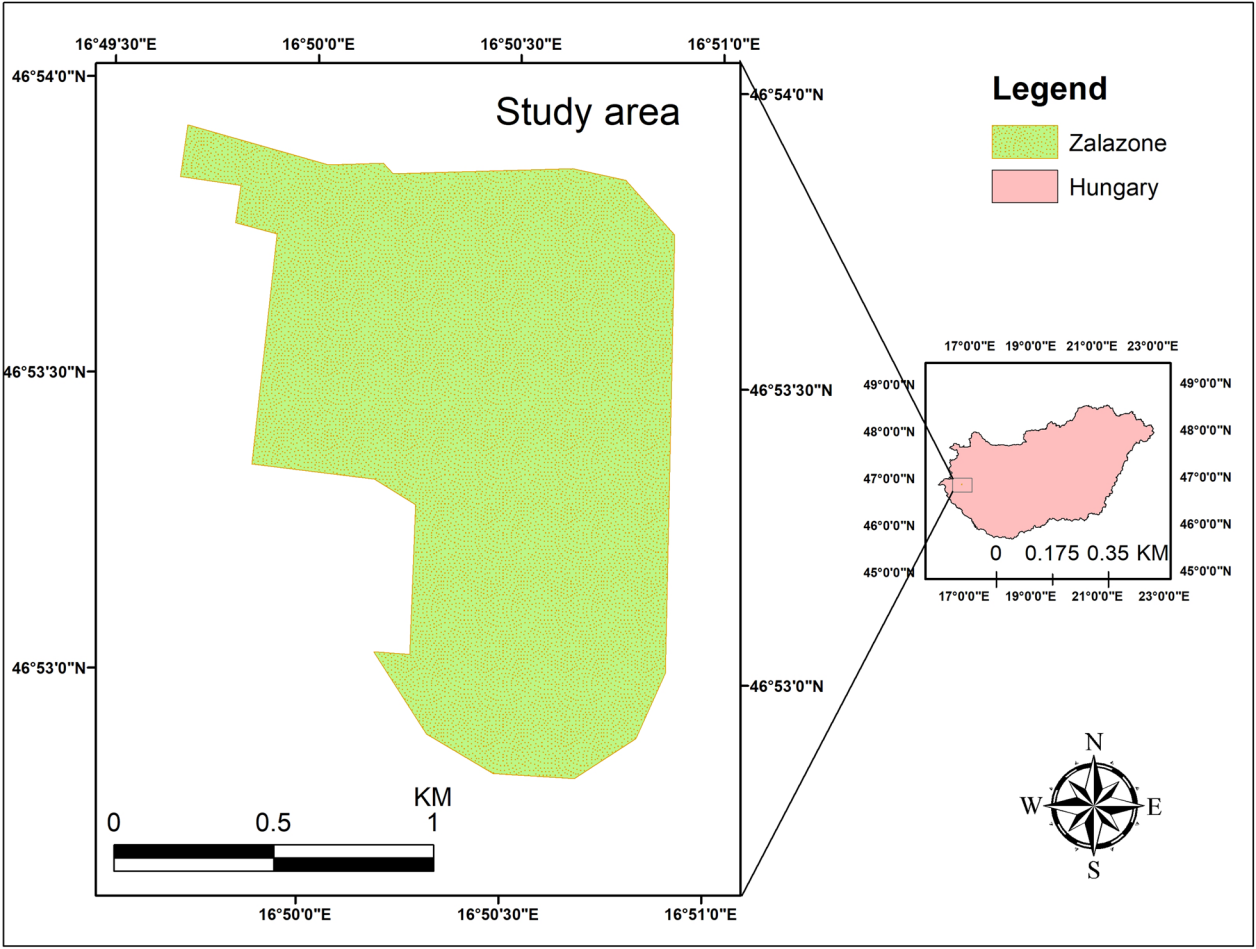
Study area (Source: authors)

ZalaZone is a long-term project in the Zala county of Zalaegerszeg, Hungary, located at 46.88947 N and 16.83676 E. It is serving as a testing ground for conventional, partially autonomous, and fully automated vehicles while also aiming to accelerate the development of AVs as illustrated in Fig. 1.

**Fig. 2:**
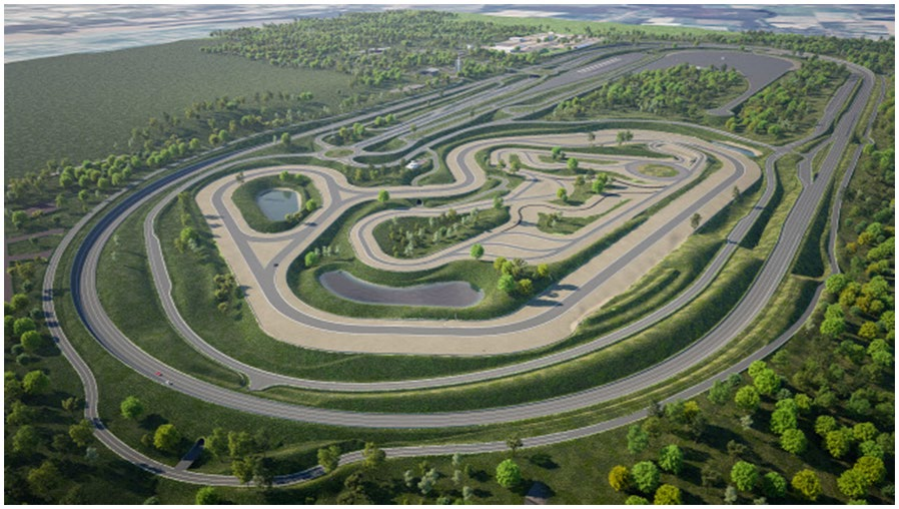
An aerial view of the ZalaZone road network (Source: CEOI)

It is a smart city-like area that includes additional environmental, traffic, network technologies, and vehicle dynamics aspects to provide realistic traffic conditions in a constrained location. A variety of lane numbers, types, and configurations, as well as the road’s surface and geometry, have all been incorporated to represent the urban setting better and realistically as presented in Fig. 2.

### Data sources and methods of analysis

The study used a microsimulation methodology to determine the impacts of AVs on road traffic emissions for a specific network. Eclipse SUMO is employed as the microsimulation program, as previously stated. DLR’s Eclipse SUMO open-source microsimulation software comes with features for measuring emissions and analyzing vehicle behavior (Lopez et al. 2018). Its versatility in modeling driver and vehicle behavior, along with its technical computing and simulation modeling capabilities, make the package suited to this article. It also provides access to a running road traffic simulation, as well as the ability to retrieve values of simulated objects and manipulate their behavior in real-time.

#### Car following model

The car-following model used by Eclipse SUMO is a modified version of Krauss’ original concept. The first Krauss car-following model was developed by Stefan Krauss in 1997. The concept of “safe speed” serves as the foundation for this model. The safe speed is determined as follows:

$$v_{{safe}} = v_{l} (t) + \frac{{g_{n} (t) - v_{l} (t)T}}{{T + \frac{{v_{f} (t) + v_{l} (t)}}{{2b}}}}$$

where ${v_l}\left(t\right), {v_f}\left(t\right), {g_n}\left(t\right), T$ and *b* stand for the speed of the leading vehicle at time t, the speed of the following vehicle at time t, the gap between the leading and following vehicle at time t, the reaction time of the driver, and the maximum deceleration, respectively. The ${v}_{safe}$ equation ensures vehicle safety. The safe speed, however, could exceed the posted speed limit or go higher than the maximum speed that a vehicle is capable of. As a result, the desired speed term is developed, which may be calculated as follows:

$${{v}_{desired}}=Min({v_f}\left(t\right)+at, {{v}_{safe}}, {{v}_{limit}})$$

where $a, t, {v}_{limit}$ indicate acceleration, time, and legal posted speed limit. The ${v}_{desired}$ equation addresses all relevant issues, including vehicle capability, law enforcement, and safety. The desired speed is specified as the lowest of the three speed settings. SUMO has a parameter for imperfection that it uses to mimic a human driver. But to achieve variation in spacing, this parameter is randomly selected for each vehicle in a time step. As a result, the vehicle’s following speed becomes:

$${v}_{t+\Delta t}^{f}=Max\{0, {v}_{desired}-a\in \eta \}$$

where $\epsilon$ and $\eta$ indicate noise amplitude and a random number. One can obtain vehicles that are travelling at varying speeds using the equation above. In this study, the default car following model is applied to legacy vehicles. The Krauss modified model contains elements that can be customized to enable us to model AVs with different levels of automation, as will be discussed in more detail below.

The following longitudinal maneuvering characteristics were taken into consideration while modeling AVs using Krauss’ modified model:

- Mingap: the offset to the leading vehicle when standing in a jam (m)
- Accel: the acceleration ability of vehicles of this type m/s^2^
- Decel: the deceleration ability of vehicles of this type (m/s^2^)
- Emergency Decel: the maximum deceleration ability of vehicles of this type in case of emergency in m/s^2^.
- Sigma: the driver imperfection (between 0 and 1).
- Tau: the driver’s desired (minimum) time headway (reaction time) (in s)

For legacy vehicles, all parameters were left at their default settings, except for the emergency deceleration, which was reduced from 9 to 8 m/s^2^. Deceleration and emergency deceleration values were 4.5 m/s^2^ and 8 m/s^2^, respectively, for all automation levels, including legacy vehicles (Kudarauskas 2007).

Apart from deceleration and emergency deceleration, all the other parameters are tuned based on the data taken from Atkins Ltd report (Atkins 2016). The report has been utilized as the primary source for level 2 and 5 AVs. Given the legacy vehicle's parameter defined above, the corresponding parameters of level 1 AVs were taken as the averaged value of legacy and level 2 AV’s as it lies between them. Also, the parameters for the remaining level 3 and 4 AVs were obtained by linearly modifying the respective data between level 2 and level 5 AVs. However, since the level 4 and 5 AVs do not require human drivers, the driver imperfection (sigma) was set to 0. Given that human interference reduces as automation levels rise, the sigma values for the various levels of automation are expected to fall linearly from the default 0.5 for legacy vehicles to 0.2 for level 3 AVs. The resulting sigma values for levels 1, 2, and 3 AVs were 0.4, 0.3, and 0.2, respectively as summarized in Table 1.

**Table 1:** The parameters for car following for varying levels of automation

Parameters	Level 0	Level 1	Level 2	Level 3	Level 4	Level 5
Mingap (m)	2.5	2	1.5	1.17	0.83	0.5
Accel (m/s^2^)	2.6	3.05	3.5	3.6	3.7	3.8
Emergency decel (m/s^2^)	8	8	8	8	8	8
Sigma (driver imperfection)	0.5	0.4	0.3	0.2	0	0
Tau (s)	1	0.95	0.9	0.8	0.7	0.6

**Fig. 3:**
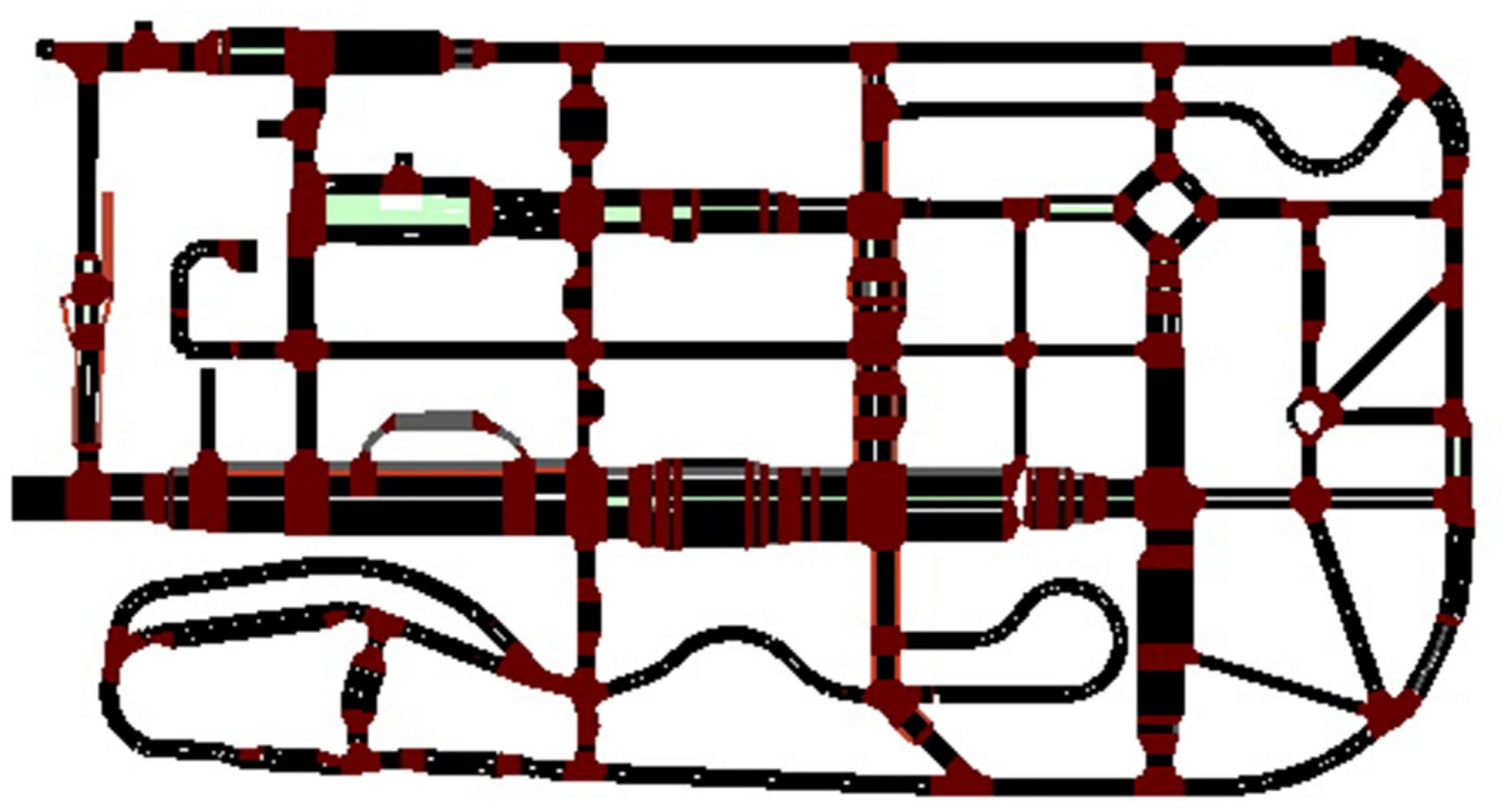
Network of ZalaZone in SUMO (Source: authors)

Like the other traffic simulation software, the road network in SUMO is made up of nodes and edges. The network presented in the research has a total length of edges of 6.42 km. There are 354 edges and 164 nodes in the network. Because all the links in the networks are presumed to be urban roads, the speed limit was set at 50 km/h. The most common traffic situations in an urban setting are stop-and-go, braking, and deceleration of vehicles during trips. These situations are achieved by utilizing traffic lights, roundabouts, and junctions that were built in ZalaZone road network as shown in Fig. 3.

#### Market penetration rates of AVs

The adoption of AVs would be hindered by a lack of technological availability and users’ lack of confidence on the technology’s safety and security. This will lead to a gradual integration of AVs into the current road networks, and that paves a way for mixed traffic in which AVs share the road with legacy vehicles.

Since the purpose of this study is to examine the impact of various levels of AV fleets on road traffic emissions, a straightforward technique has been employed to consider AVs’ capabilities and their market penetration rates. This technique is based on the fundamental and well-known premise that the deployment of AV fleets should be a gradual process and ought to start from driver assistance, even though it’s theoretically possible to leverage full automation.

Limiting the technical capacities to driver assistance would provide the ground to efficiently and effectively improve the systems based on users’ feedback and increase the levels. It’s also important to prevent the cataclysmic effect that results from technological uncertainty or full implementation of the theory. On the other hand, starting from driver assistance then giving full control of driving tasks to the system helps to gain users’ trust in the technology over time by providing them with the most demanding assistance in difficult driving conditions. The assumptions mentioned along with climate policy and strategy create a conducive environment to increase the level of automation of AV fleets and their market penetration rates (Atkins 2016).

**Fig. 4:**
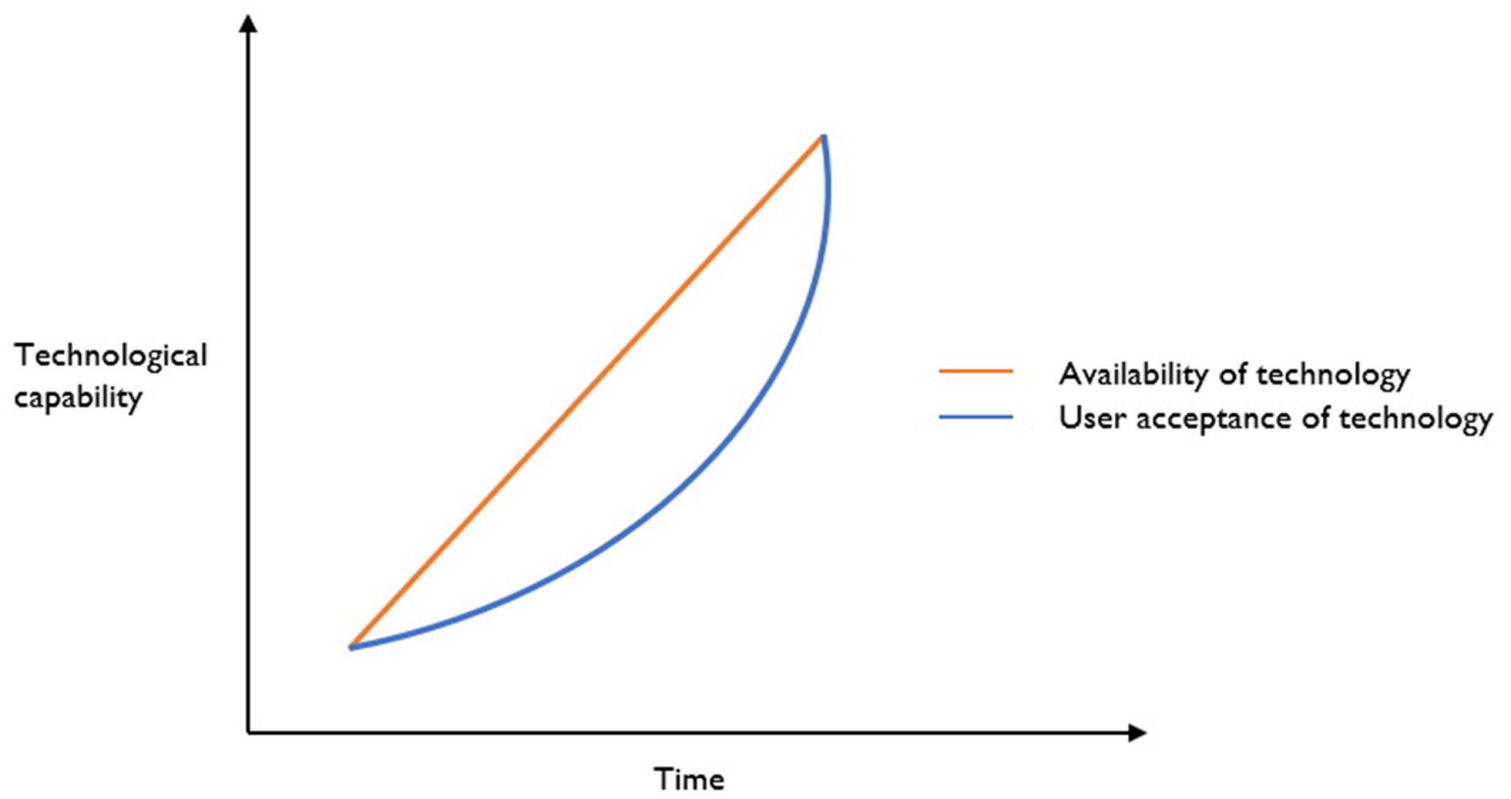
Future states of availability and user acceptance (Source: (Atkins 2016))

Figure 4 presented graphically with how the technological complexity of AVs will develop over time. The fact that the technology is readily available in this circumstance does not guarantee that users will find it acceptable. Early adopters typically open the door to innovative technologies before they are broadly accepted. This is reflected in the market penetration rates for varying levels of automation.

The ratio of AVs to legacy vehicles in the scenarios increases along with the level of automation. As demonstrated in Table 2, the technological capabilities of each vehicle type in the AV fleets vary to match the user’s choices and confidence in the technology. The phrase “upper bound” is used to describe the fifth scenario, in which all vehicles are totally automated. The modeled situations are presented in Table 2.

**Table 2:** AV market penetration rates

No	Scenarios	The ratio of legacy vehicles (%)	Automated vehicles				
			Level 1 (%)	Level 2 (%)	Level 3 (%)	Level 4 (%)	Level 5 (%)
1	Base	100	0	0	0	0	0
2	25% penetration	75	15	5	5	0	0
3	50% penetration	50	25	10	10	5	0
4	75% penetration	25	25	20	15	10	5
5	100% penetration	0	15	20	20	25	20
6	Upper bound	0	0	0	0	0	100

#### Emission modeling

Emissions are calculated using the Eclipse SUMO HBEFA3-based emission model. This model was developed by collecting relevant information from the HBEFA database and fitting it to a continuous function. The function was obtained by simplifying the power required by the engine to overcome the external load (engine energy consumption rate) as incorporated in Eclipse SUMO:

$$P={c}_{0}+{c}_{1}va+{c}_{2}v{a}^{2}+{c}_{3}v+{c}_{4}{v}^{2}+{c}_{5}{v}^{4}$$

where ${c}_{n}$ are constant parameters, a is acceleration and v is speed. To compute the power demand, the emission factors are selected from the HBEFA database, and the coefficients are determined based on the type of vehicle and engine used by the vehicles.

#### Simulation trials

The simulation period is 21,600 s (about 6 h), and scenarios with various levels of AVs penetration were used to simulate the network. The penetration rates for AVs began at 0% and increased by 25% until they hit 100% in the fourth scenario. The last scenario similarly has 100% AVs; however, they are all level 5 vehicles. Six different types of vehicles are modeled, starting from type 0 to type 5. All of them are Euro Norm 5 vehicles with gasoline engines, but they have varying levels of technical capabilities to mimic the levels of automation. Those vehicles are defined by specific parameters that indicate their unique behaviors at the respective levels. The vehicles named as type 0, are legacy vehicles, and type 1 represents level 1 AVs, and similarly, all the way up to type 5, which stands for level 5 AVs, or fully automated vehicles.

Two demand situations are considered with the objective of understanding how vehicles’ emission behavior alters under different traffic conditions:

- A “peak” period model, in which model is characterized by congestion, queuing, delays and low traffic speeds.
- A “non-peak” period model, where vehicle speeds are close to the allowed speed or the legal speed limits are predominantly maintained by vehicles.

**Fig. 5:**
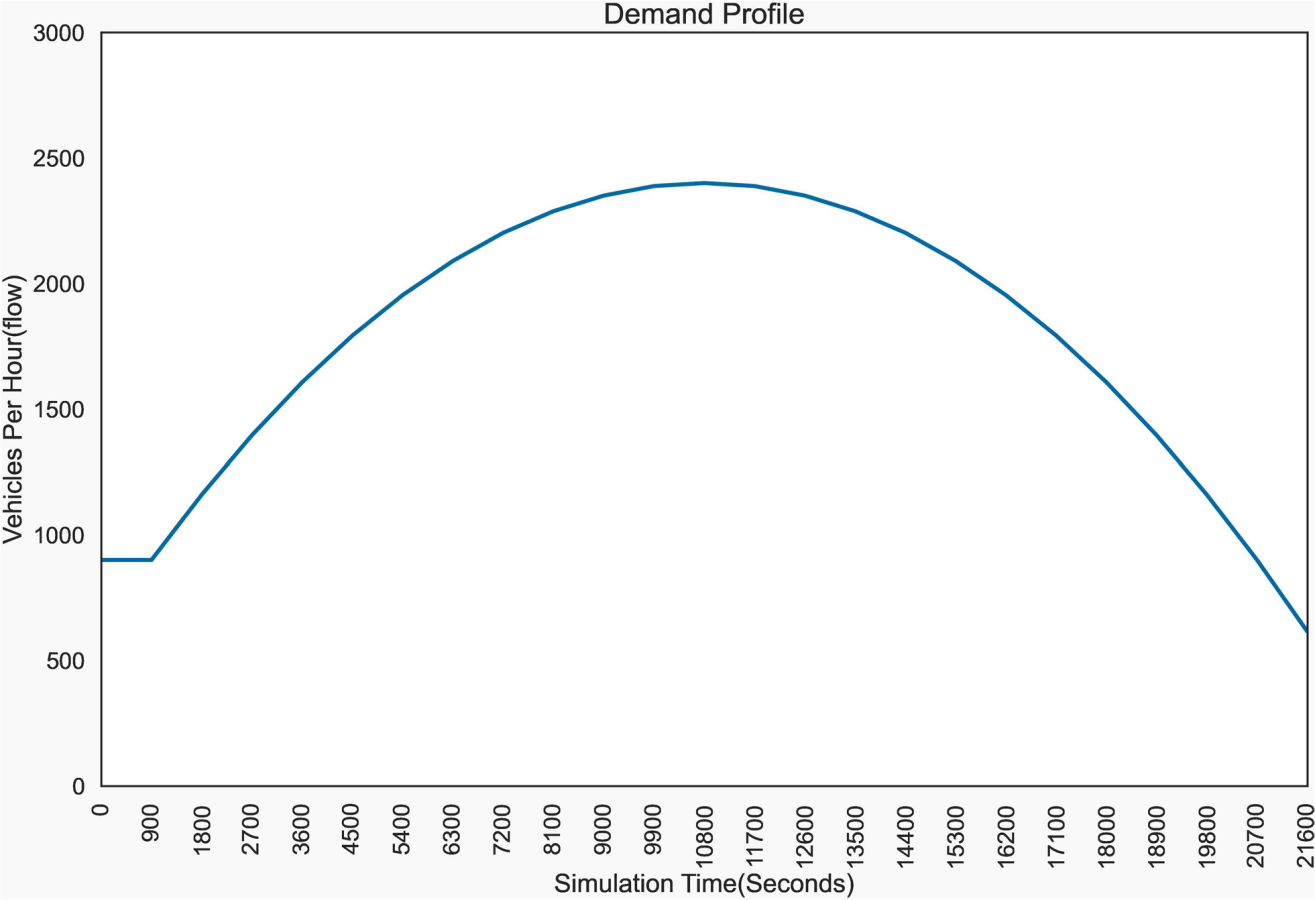
Demand profile (Source: authors)

In this study the vehicles running from 9900 to 12,800 simulation time were considered to investigate emissions under the peak hour traffic situations whereas the vehicles on the network from 0–900 s for non-peak hours, as shown in Fig. 5.

## Results and discussions

This study looked at six parameters to investigate the impact of varying levels of AVs on road traffic emissions, five of which are pollution types and fuel consumption. They are assessed and summarized for the base model and five other scenarios. The scenarios were developed by altering the number and level of AVs on the network with reference to the base model. Additionally, accelerations for all types of vehicles were measured during congested “peak” demand periods and uncongested “non-peak” demand periods to track the emission behavior of AVs under various traffic conditions and study the dependency of emissions on acceleration.

### A vehicle's emissions behavior

It is vital to understand the emission behavior for each type of vehicle separately to see their impact on the network-level aggregated emissions. A 22 km (approximately 13.67 mi) route was defined by rerouting the designated vehicles on the network to get attributes that can be used to easily distinguish their emission behaviors. While the designated vehicles with various levels of automation were running along that route, the relevant information was gathered and analyzed. To comprehensively observe and understand the emission behavior under various traffic conditions, vehicles are also simulated for peak and non-peak hours. As acceleration is one of the key factors affecting a vehicle’s emissions behavior, a couple of acceleration-related data are analyzed and described as follows.

#### Correlation between acceleration and emission

**Fig. 6:**
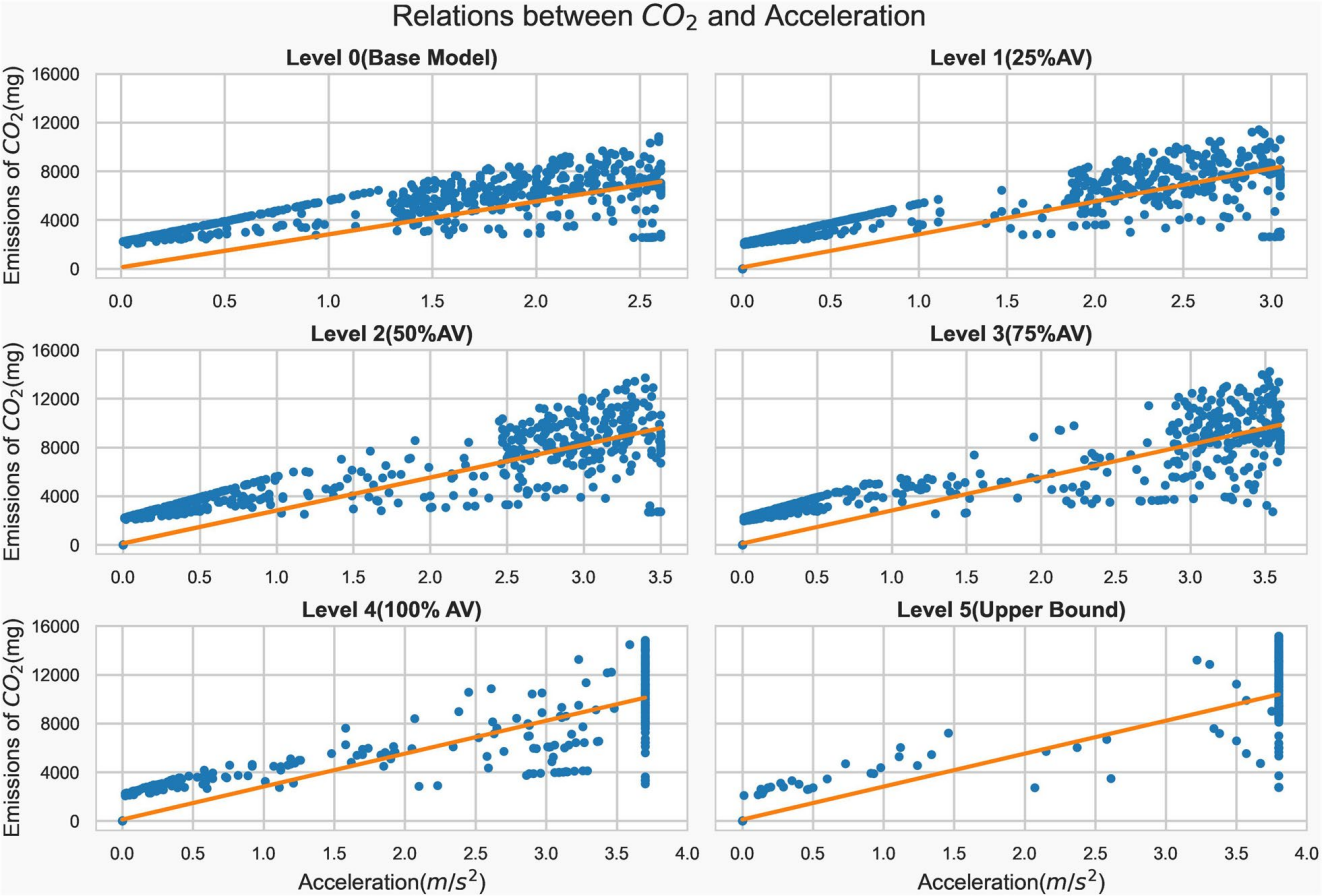
Carbon dioxide emissions and acceleration scatter plots (Source: authors)

Using the Pearson correlation method, the relationship between acceleration and emission is computed. The computation was carried out for all vehicle types. The scatter graphs were plotted by using a vehicle type per scenario and CO_2_ emissions, which is considered representative emission. The results were significantly positive and have strong linear correlations, with a range of 0.7 to 0.90. According to Fig. 5, the correlation coefficient for legacy vehicles under the base model is 0.76, whereas for fully automated vehicles under the upper bound scenario is 0.93. Furthermore, as the level of automation increases the correlation coefficient also increases. It is 0.83, 0.87, 0.88, and 0.92 for level 1, level 2, level 3, and level 4 vehicles, respectively. The maximum acceleration value and its corresponding accelerating capabilities of the vehicles increase as the automation levels increase. So, the maximum accelerations for legacy vehicles and level 5 AVs were 2.6 m/s^2^ and 3.8 m/s^2^, respectively as depicted in Fig. 6.

### A vehicle's acceleration rate

**Fig. 7:**
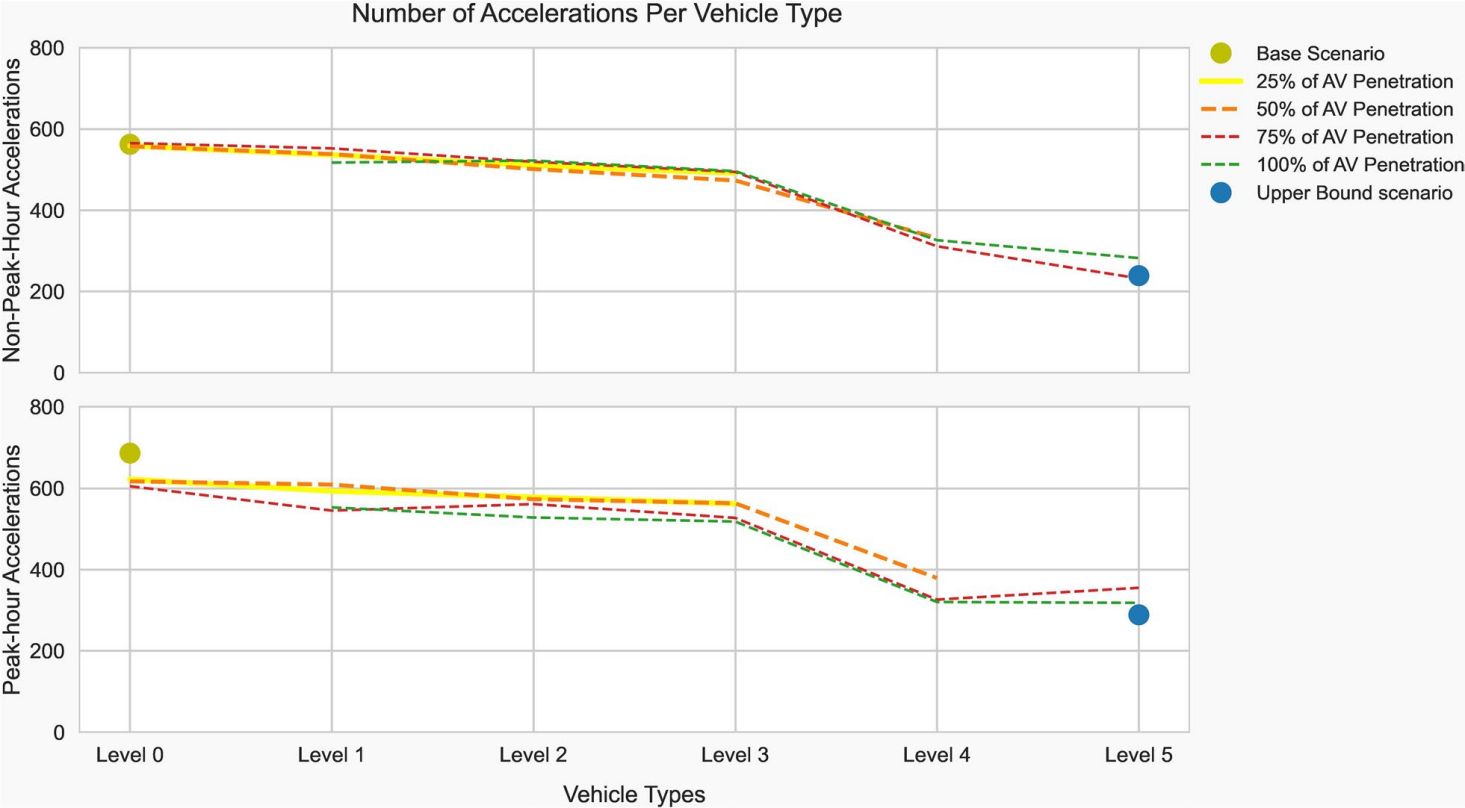
The total number of vehicle accelerations

Because of the strong relationship between acceleration and emissions, the number of accelerations each vehicle type makes over the length of a 22-km trip must be counted and analyzed to estimate their influence on emissions. Since “on-demand” traffic situations expose vehicles to a more stop-and-go movement than normal flow, the simulation was carried out during peak and off-peak hours. For instance, the legacy vehicle accelerated 125 times more during peak hours than it did during non-peak hours (687 versus 562 times). As the level of vehicle automation rises, less acceleration is conducted by the respective vehicles, as shown in Fig. 7. Given the low penetration rate of AVs, a level 3 automated vehicle accelerates 491 times during off-peak hours and 562 times during peak hours. In the upper bound case, a level 5 vehicle accelerated 239 and 289 times during off-peak hours and peak hours, respectively, which is approximately 57% fewer accelerations than the base model’s legacy vehicles.

### Emissions by vehicle type

The pollutants emitted from each type of vehicle must be measured to better understand their emission behavior. Under the scenarios that have been predefined, the emissions for all vehicle types were collected and summarized. When compared to the base model, the upper bound scenario’s peak hour emissions were reduced by 48%. However, there was no substantial variation in emissions between various vehicle types during off-peak hours.

**Fig. 8:**
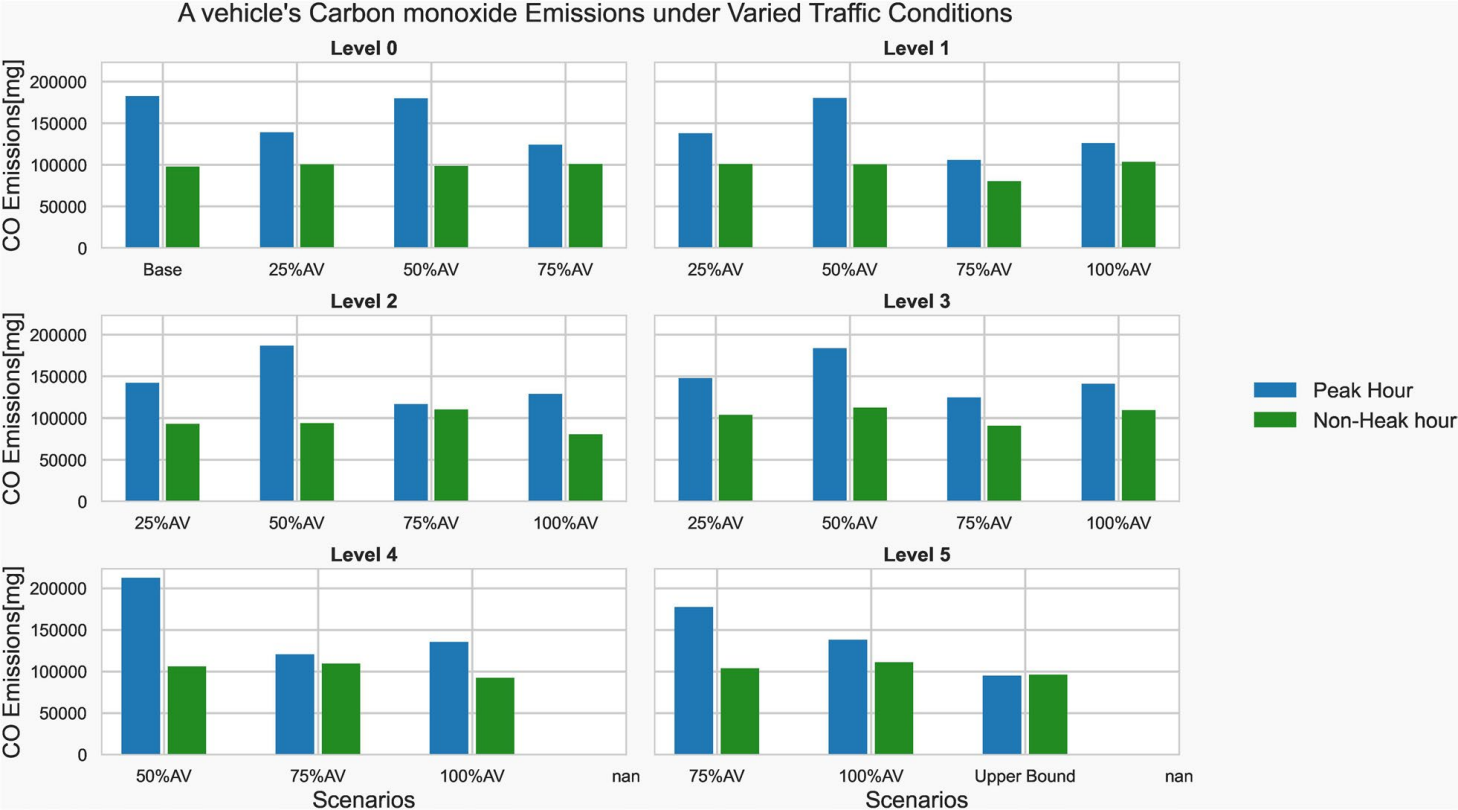
Individual vehicle emission levels

Figure 8 demonstrates that, even during peak hours, there was no consistent reduction in emissions as vehicle automation increases. Given the mixed traffic, there was no significant reduction in emissions. For instance, with a 50% AV penetration rate, vehicle emissions have gotten worsened. The major conclusion is that in a mixed traffic situation, the combined effect of flow disruption by legacy vehicles with AVs’ technical capabilities to quickly attain the top speed, which has a significant positive relationship with emissions, will likely increase the emissions. According to the scatter plot in Fig. 4, the vehicles with significantly larger acceleration values are also the ones with a higher level of automation.

The difference in emissions between peak and non-peak hours shows that the net difference in emissions between those traffic conditions was larger for legacy vehicles. On the other hand, network traffic conditions had no impact on fully automated vehicles.

The time it takes for a vehicle to accelerate and decelerate gets shorter as the vehicle’s automation level rises or human interference plays less of a role. As acceleration capability increases, emissions also increase because of their direct correlation. On the other hand, the rate of a vehicle’s acceleration drops as automation levels increase. Despite having a higher accelerating capability, it has been found that the emissions reduction of AVs was realized by a lower rate of acceleration conducted during the trip.

### Aggregated emission

To fully understand how the deployment of AVs impacts emissions from road traffic, it is necessary to look at network-level emissions as well as emissions from individual vehicles. Five scenarios are developed based on the base model to investigate their effects. For comparison purposes, the absolute values of all potential pollutants from vehicles over the simulation period are obtained, aggregated, and summarized for each scenario.

Table 3 summarizes the improvements for each scenario in comparison to the baseline model. The result shows that as AVs deployment increases, vehicle fuel consumption improved. This leads to positive trends in the reduction of other environmentally harmful pollutants. At 25% AV penetration with limited capabilities, CO, HC, PM_x_, and NOx all dropped by more than 14%, 13%, 10%, and 8%, respectively. Both fuel consumption and CO_2_ emissions fell by 8.35%. When AVs penetration reached 50%, of which 35% were level 1 and level 2 vehicles, CO emissions decreased by 17% from the reference base model, while HC and PM_x_ emissions decreased by 16% and 12%, respectively.

**Table 3:** The percentage reduction in emissions in comparison with the base model

Scenarios	CO	CO_2_	HC	PM_X_	NOx	Fuel consumption
Base	–	–	–	–	–	–
25%AV	− 14.17%	− 8.35%	− 13.38%	− 10.29%	− 8.54%	− 8.35%
50%AV	−17.26%	− 10.11%	− 16.31%	− 12.42%	− 10.33%	− 10.11%
75%AV	− 27.74%	− 15.74%	− 26.19%	− 19.96%	− 16.29%	− 15.74%
100%AV	− 35.22%	− 18.55%	− 33.23%	− 25.41%	− 19.81%	− 18.55%
Upper bound	− 38.56%	− 17.09%	− 36.30%	− 28.12%	− 19.78%	− 17.09%

**Fig. 9:**
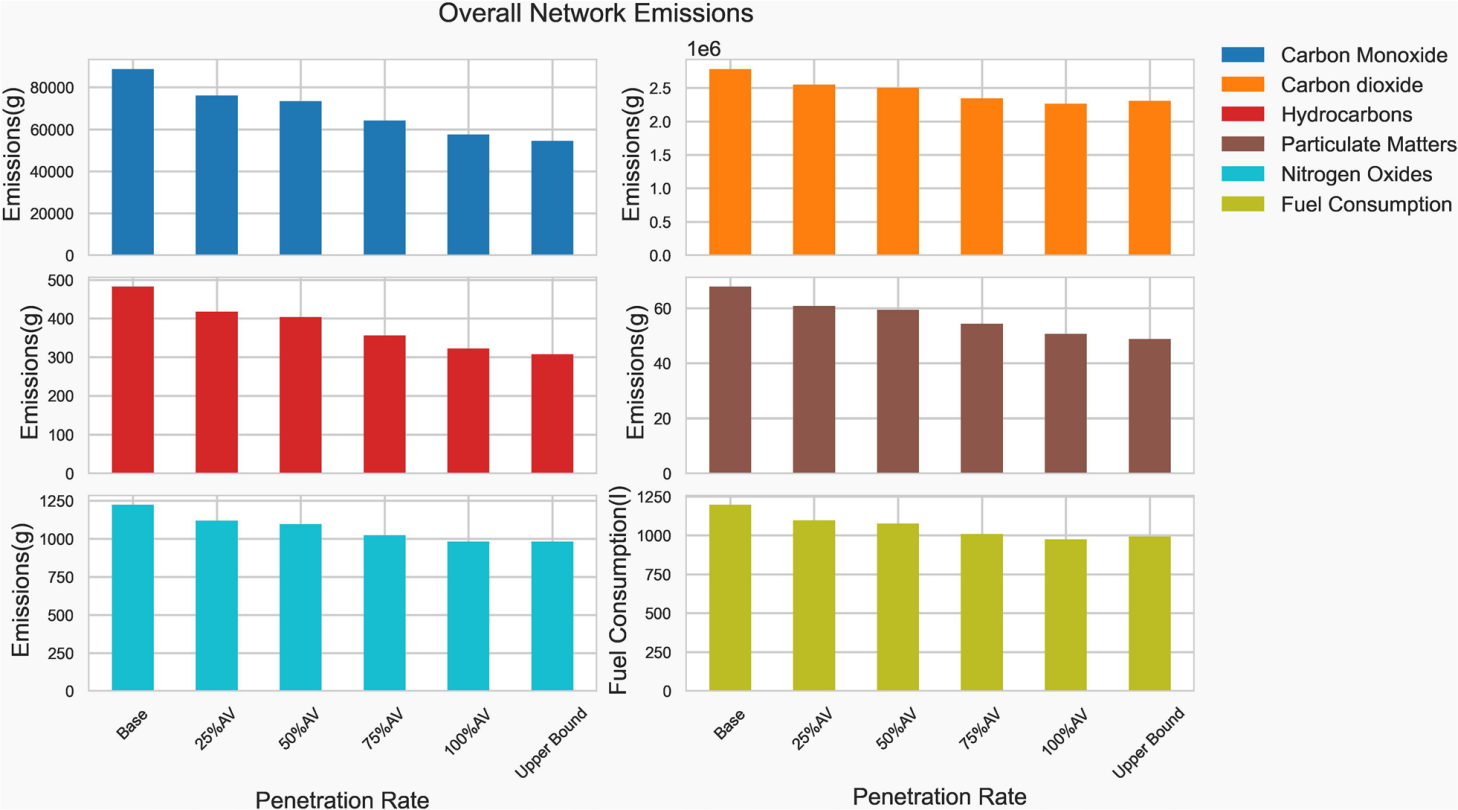
Overall emissions from the network (Source: authors)

The reduction in emissions continued up to the upper bound scenarios, which represent 100% penetration of fully automated vehicles that behave “aggressively,” including closely following other vehicles, accepting narrower gaps, and with no driver imperfections. Emissions of CO, HC, and PM_X_ were reduced by 39%, 36%, and 28%, respectively, under the upper-bound scenario as presented in Fig. 9.

The main conclusion is that the widespread deployment of automated vehicles has the potential to greatly reduce traffic-related emissions, especially if smooth driving is practicable.

## Conclusion

Climate change and air pollution have come to the forefront of our society and the government’s concerns. The concerns regarding rising world temperatures, increasing emission levels, and over-exploitation of natural resources draw stakeholders’ attention and compel officials to take preventive measures to reverse humanity’s destructive effects on the environment. In terms of environmental pollution caused by the transportation sector, road transport has emerged as the primary emitter. Numerous stakeholders (automakers, community managers, municipalities, and vehicle operators etc…) are now involved in the process of mitigating the negative effects of mobility on the ecosystem through a variety of mechanisms. Several proposed alternatives include vehicle automation, riding a bike or walking instead of driving, taking public transport, carpooling or car sharing, and having a fuel-efficient vehicle.

Vehicle automation is believed to play a significant role to resolving the catastrophe that contemporary societies face because of traffic problems. Most automobile manufacturers are engaged in fierce competition to introduce entirely self-driving and hybrid vehicles to the market. Additionally, this study examined how much automated vehicles are likely to contribute to mitigating air pollution, besides the other advantages that will accrue from them, such as reducing road accidents, avoiding congestion, rising the quality of service provided by infrastructures, and fuel savings. This study is aimed at examining the effect of automated vehicles on pollutant emissions under various scenarios. Even though the emission from vehicles is often dependent on their geometrical and physical properties, for the sake of simplification, all vehicles used in this simulation are completely similar except for their maneuvering capability. The effects evaluation of vehicle weight reduction along with vehicle automation on pollution reduction is being deferred for further studies.

The analysis was conducted using the Simulation of Urban Mobility (SUMO) software. The primary features that distinguish automated from conventional vehicles are car-following and lane-change behaviors. The Krauss modified and LC2013 were used as car-following and lane-changing models, respectively. The levels of automation are achieved by adjusting the car’s following parameters. The numerous emission models were checked, and although some of them have the needed emission measurement kits, they are either not supported by SUMO or commercial packages. As a result, HBEFA v3.1 was used in this study to measure pollution.

Due to technical difficulties and user skepticism about technology in terms of safety and security, it is impractical to replace all existing legacy vehicles with AVs. Given the above information, AVs were gradually introduced to the network over the course of five consecutive scenarios. Each scenario has a unique mix of legacy and varying levels of automated vehicles. Additionally, every possible traffic condition that might occur on the real road network was considered.

Correlation analyses have revealed a strong linear relationship between vehicle acceleration and emissions. It increases with the level of automation. The ability of AVs to swiftly accelerate makes the relationship significant compared to legacy vehicles. The correlation coefficient for legacy vehicles was 0.76, whereas level 5 AVs had 0.93. On the other hand, the vehicles’ automation level and acceleration rates are inversely proportional. The rate of acceleration conducted by the level 5 AVs was approximately 57% lower compared to legacy vehicles during the same trip. Besides automation levels, traffic conditions also influence the rate of acceleration. During peak hours, the legacy vehicles performed 125 times more acceleration than during non-peak hours, which was the same across various vehicle types. Despite having a higher acceleration capability, it has been found that the emissions reduction of AVs was realized by a lower rate of acceleration during the trip.

The modeling results showed that AVs have the potential to substantially cut the amount of pollution emitted by road traffic. In the optimistic case (i.e., when all vehicles are replaced by fully automated vehicles), AVs reduce emissions of carbon monoxide (CO) by 38.56%, carbon dioxide (CO_2_) by 17.09%, hydrocarbons (HC) by 36.3%, particulate matter (PM_x_) by 28.12%, and nitrogen oxides (NOx) by 19.78%.

## Data Availability

The datasets used or analyzed during the current study are available from the corresponding author upon a reasonable request.

## Abbreviations

ARTEMIS: Assessment and Reliability of Transport Emission Models and Inventory Systems
AVs: Automated vehicles
CAV: Connected autonomous vehicles
CO: Carbon monoxide
CO_2_: Carbon dioxide
COPERT: Computer Program to Calculate Emissions from Road Transport
COVID 19: Coronavirus Disease of 2019
CV: Conventional vehicles
DLR: Deutsches Zentrum für Luft- und Raumfahrt (German Aerospace Center)
EEA: European Economic Area
EMEP: European Monitoring and Evaluation Program
EPA: Environmental Protection Agency
ERMES: European Research on Mobile Emission Source
HBEFA: Handbook Emission Factors for Road Transport
HC: Hydrocarbons
JRC: Joint Research Centre of the European Commission
LC2013: Lane Changing Model 2013
LV: Legacy vehicles
MOVES: Motor Vehicle Emissions Simulator
NOx: Nitrogen oxides
PHEM: Passenger Car and Heavy-Duty Emission Model
PM_x_: Particulate matter
PM_2.5_: Particulate matter (particles with diameter of 2.5 micrometers or less)
SUMO: Simulation of urban mobility
VOCs: Volatile organic compounds
VSP: Vehicle specific power
WHO: World Health Organization
